# Construction of a Three-Dimensional Culture Model of HSV-1 Based on the Nano-Self-Assembling Peptide RADA16-I and Preliminary Exploration of the Relationship Between HSV-1 and Autophagy

**DOI:** 10.3390/microorganisms14030601

**Published:** 2026-03-08

**Authors:** Zhen Hu, Yun-E Xu, Jie Zhang, Xue Luo, Jia-Zhe Li, Yu-Tong Wang, Heng-Mei Li, Xin Sun, Sheng-Yu Wang, Hong Song, Di-Shu Ao

**Affiliations:** Department of Microbiology, School of Basic Medical Sciences, Zunyi Medical University, Zunyi 563000, China; hz197432@outlook.com (Z.H.); yunexu@zmu.edu.cn (Y.-E.X.); 13560437669@163.com (J.Z.); 18188198602@163.com (X.L.); 18198031581@163.com (J.-Z.L.); wongmiekott@foxmail.com (Y.-T.W.); 18183422187@163.com (H.-M.L.); coolxin@foxmail.com (X.S.); sywang2012@126.com (S.-Y.W.)

**Keywords:** RADA16-I, HSV-1, 3D culture, Vero cell, autophagy

## Abstract

Herpes simplex virus type 1 (HSV-1) is a neurotropic alphaherpesvirus that interacts dynamically with host cells within structured tissue environments. Conventional two-dimensional (2D) cultures do not fully recapitulate these spatial and microenvironmental features. In this study, we established a three-dimensional (3D) culture system using the self-assembling peptide RADA16-I to generate an extracellular matrix–mimetic hydrogel scaffold. This platform supported the formation of stable Vero cell spheroids that remained viable for more than 30 days. Following HSV-1 infection, viral spread initiated at the spheroid periphery and progressively extended toward the core. Sustained viral replication was detected for up to 22 days, indicating long-term maintenance of infection within the 3D structure. Ultrastructural examination identified viral particles and vesicular compartments consistent with autophagy-related organelles. Comparative analysis of autophagy-associated markers revealed distinct temporal patterns between 2D monolayer cultures and 3D spheroids. In the 3D system, LC3B-II levels progressively increased, accompanied by a reduction in p62, suggesting altered regulation of autophagic flux relative to conventional 2D conditions. These findings demonstrate that the RADA16-I-based 3D culture model supports prolonged HSV-1 infection and reproduces key spatial features of viral dissemination. The differential autophagic responses observed between 2D and 3D systems highlight the influence of cellular architecture on host–virus interactions and support the application of 3D culture platforms for mechanistic studies of HSV-1 pathogenesis.

## 1. Introduction

Herpes simplex virus type 1 (HSV-1) is a neurotropic alphaherpesvirus with a linear double-stranded DNA genome of approximately 150 kbp. Clinically, HSV-1 infection manifests as gingivostomatitis, keratoconjunctivitis, and encephalitis, and may result in irreversible blindness, permanent neurological sequelae, or death in severe cases [1]. Following primary infection, HSV-1 establishes lifelong latency in sensory neurons, particularly within the trigeminal ganglion. Periodic reactivation, often triggered by immunosuppression or stress, leads to recurrent disease [2]. The combination of neuroinvasiveness, latency, and reactivation underscores the need for in vitro models that can recapitulate the neural microenvironment and support long-term infection dynamics.

Most mechanistic studies of HSV-1 have relied on two-dimensional (2D) cultures of primary neurons or immortalized cell lines. These systems have substantially advanced our understanding of viral entry, replication, latency-associated pathways, and antiviral drug screening [2]. However, 2D cultures lack the architectural complexity of native tissues. They do not reproduce key features of the three-dimensional (3D) microenvironment, including extracellular matrix (ECM) stiffness, spatial cell polarity, cell–cell interactions, and paracrine signaling networks. Moreover, 2D systems are limited in their ability to model prolonged biological processes such as persistent or latent infection [3]. In recent years, 3D culture systems have emerged as valuable platforms for modeling tissue complexity and host–pathogen interactions. For example, a Matrigel-based 3D model of uveal melanoma has been used to investigate HSV-1–tumor cell interactions [4]. However, Matrigel, as an animal-derived matrix, has an undefined composition, batch-to-batch variability, and potential risks of pathogen contamination, all of which limit reproducibility and standardization [5]. Alternative synthetic scaffolds have also been explored. An acrylate-based transparent methyl methacrylate system was used to construct a 3D fibroblast model and revealed slower recovery of infected cells in 3D compared with 2D culture, suggesting microenvironment-dependent modulation of infection responses [6]. Nevertheless, complex fabrication procedures and contamination susceptibility restrict its broader application.

The self-assembling peptide RADA16-I has emerged as a promising scaffold material for 3D cell culture. Composed of a well-defined amino acid sequence, RADA16-I exhibits excellent biocompatibility, minimal immunogenicity, and high batch-to-batch consistency [7]. Under physiological conditions, it self-assembles into a nanofibrous hydrogel network with water content exceeding 99%, closely mimicking the structural and permeability characteristics of native ECM and facilitating diffusion of nutrients and signaling molecules [8]. Its biomedical applications continue to expand, including use as a nanogel carrier to protect bioactive peptides from enzymatic degradation [9], as a drug delivery platform in tumor immunotherapy [10], and as a scaffold for promoting axonal regeneration and neuronal growth in neuroscience research [11]. Our group previously demonstrated that a RADA16-I-based 3D culture system supports long-term adenovirus infection, highlighting its potential utility in virological modeling [12].

Despite advances in 3D infection models, most studies have focused primarily on model construction and phenotypic characterization, with limited investigation into host regulatory pathways during infection. Recent work using human induced pluripotent stem cell (iPSC)-derived brain organoids has provided important insights into HSV-1-induced neuroinflammation and injury. For example, integrated single-cell transcriptomic and immunohistochemical analyses revealed robust activation of the TNF–NF-κB signaling pathway following HSV-1 infection [13]. Studies in 3D neurovascular models further demonstrated suppression of autophagic flux in glial cells during HSV-1 infection, suggesting a potential role for autophagy in viral neuropathogenesis [14]. Autophagy (macroautophagy) is an evolutionarily conserved, lysosome-dependent process responsible for degrading damaged organelles, misfolded proteins, and invading pathogens. During this process, cytoplasmic cargo is sequestered into double-membrane vesicles termed autophagosomes, which subsequently fuse with lysosomes for degradation and recycling [15,16]. To date, most investigations of HSV-1–autophagy interactions have been conducted in 2D cell cultures, which do not adequately reflect the influence of tissue architecture on cellular responses. Comparative analyses have demonstrated significant differences in transcriptional profiles and cytopathic effects between 2D and 3D HSV-1 infection models [17], emphasizing that culture dimensionality is a critical determinant of virus–host interactions.

Based on these considerations, the present study aimed to establish a RADA16-I-based 3D culture system for modeling HSV-1 infection in vitro under conditions that more closely approximate the physiological microenvironment. We further compared autophagy dynamics between 2D and 3D culture systems following HSV-1 infection. In 2D cultures, autophagy exhibited a biphasic pattern characterized by early suppression, transient activation, and subsequent inhibition. In contrast, the 3D system displayed relative quiescence during early infection, followed by marked enhancement of autophagic activity at later stages. These findings suggest that cellular architecture and microenvironmental context substantially influence the temporal regulation of autophagy during HSV-1 infection. Further mechanistic studies using higher-resolution approaches will be required to elucidate the underlying regulatory pathways.

## 2. Materials and Methods

### 2.1. Scanning Electron Microscope (SEM) Characterization

The self-assembling peptide RADA16-I (Ac-RADARADARADARADA-CONH_2_, purity ≥98%) was synthesized using the Fmoc solid-phase method and purchased from Shanghai Biotech Bioscience & Technology Co., Ltd. (Shanghai, China). A 0.5% (*w*/*v*) RADA16-I solution was prepared in 10% (*w*/*v*) sucrose.

Hydrogel samples were fixed in glutaraldehyde, dehydrated through a graded ethanol series, and subjected to critical point drying. Specimens were mounted on aluminum stubs and sputter-coated with a thin layer of metal using an ion sputter coater prior to imaging. Morphology was examined using a Hitachi S-3400N scanning electron microscope (Hitachi, Tokyo, Japan).

### 2.2. Three-Dimensional (3D) Cell Culture

Vero cells were obtained from the China Center for Type Culture Collection (CCTCC, Wuhan, China). Cells were cultured in Minimum Essential Medium (MEM; Gibco, Grand Island, NY, USA) supplemented with 10% fetal bovine serum (FBS) at 37 °C in a humidified incubator containing 5% CO_2_. When cells reached approximately 90% confluence, they were dissociated with 0.05% trypsin–EDTA and resuspended in 10% sucrose solution. For 12-well plates, 75 μL of cell suspension (3 × 10^4^ cells) was mixed 1:1 (*v*/*v*) with 1% (*w*/*v*) RADA16-I hydrogel (75 μL) and seeded into each well. Constructs were incubated at 37 °C with 5% CO_2_. Culture medium was added after 0.5, 2, and 24 h and subsequently replaced every 2 days. For 96-well plates, 25 μL of cell suspension (1 × 10^4^ cells) was mixed 1:1 with 1% RADA16-I hydrogel (25 μL) and seeded using the same procedure.

### 2.3. MTS Assay for Cell Proliferation

#### 2.3.1. Proliferation in 3D Culture

Vero cells were cultured in 1% RADA16-I hydrogel in 96-well plates. At days 0, 2, 4, 6, 8, 10, 12, 14, 16, 18, 20, 22, 24, 26, 28, and 30, 20 μL of MTS reagent (Promega, Madison, WI, USA) was added per well (*n* = 6 per time point). After incubation for 2 h at 37 °C in the dark, absorbance was measured at 490 nm using a microplate reader.

#### 2.3.2. Proliferation After HSV-1 Infection

After 3 days of 3D culture, cells were infected with HSV-1 at a multiplicity of infection (MOI) of 1 and incubated overnight at 37 °C. MTS reagent (20 μL per well; *n* = 6) was added at days 0–26 post-infection (every 2 days as described above). Absorbance at 490 nm was measured after 2 h of incubation.

### 2.4. Quantitative Cellular DNA Assay

Cell proliferation was further quantified using the CyQUANT™ Cell Proliferation Assay Kit (Invitrogen, Carlsbad, CA, USA). For baseline proliferation, gel–cell constructs were harvested at days 0–30 (every 2 days; n = 6 per time point). For post-infection analysis, cells were infected after 3 days of 3D culture (MOI = 1) and harvested at days 0–26 post-infection (n = 6). Samples were washed three times with ice-cold phosphate-buffered saline (PBS), resuspended in 50 mM sodium citrate/100 mM NaCl buffer, and stored at −80 °C. Cells were lysed by three freeze–thaw cycles. Fluorescence was measured (excitation 360 nm, emission 460 nm) using a Varioskan Flash microplate reader (Thermo Fisher Scientific, Waltham, MA, USA).

### 2.5. Cell Viability and Morphology Assessment

#### 2.5.1. Viability in 3D Culture

Vero cells were encapsulated in 1% RADA16-I and cultured in 12-well plates. On days 12 and 18, live/dead staining was performed using calcein-AM (Ca-AM), propidium iodide (PI), and DAPI (Sigma-Aldrich, St. Louis, MO, USA). F-actin cytoskeleton organization at day 18 was assessed using phalloidin staining (Sigma-Aldrich). Images were captured using a fluorescence microscope.

#### 2.5.2. Viability After Infection

After 3 days of 3D culture, cells were infected with HSV-1 (MOI = 1). At 6 and 18 days post-infection (dpi), cultures were stained with Ca-AM, PI, and DAPI and observed under a fluorescence microscope.

### 2.6. HSV-1 Infection in 3D Culture

HSV-1 (strain VR-1789) was obtained from the American Type Culture Collection (ATCC, Manassas, VA, USA). Three-day-old 3D Vero cultures were infected with HSV-1 at an MOI of 1 and incubated overnight at 37 °C. After infection, wells were washed three times with PBS and replenished with complete medium. Infection progression was monitored daily.

### 2.7. Transmission Electron Microscopy (TEM)

For TEM analysis, 2D cultures were collected at 16 h post-infection (hpi), whereas 3D cultures were harvested at 3 dpi. Samples were fixed in 1% osmium tetroxide at 4 °C for 3 h, dehydrated through a graded ethanol series, and embedded in Spurr resin. Ultrathin sections (70 nm) were cut, stained with uranyl acetate and lead citrate, and examined using a JEOL JEM-1230 transmission electron microscope (JEOL, Tokyo, Japan) at 80 kV.

### 2.8. Quantitative PCR (qPCR) for Viral Replication

Vero cells cultured in 1% RADA16-I were infected with HSV-1 (3 × 10^4^ genome copies per well). Supernatants and hydrogel-embedded cells were collected at days 0–22 post-infection (every 2 days) and stored at −80 °C.

Viral DNA was extracted using the TIANamp Virus DNA/RNA Kit (Tiangen Biotech, Beijing, China). qPCR was performed using SsoAdvanced™ Universal SYBR^®^ Green Supermix (Bio-Rad, Hercules, CA, USA) on a CFX96 Real-Time PCR system.

The primer sequences were:

Forward: 5′-ATCGGCGAGTACTGCATACA-3′;

Reverse: 5′-GAGCTCCAGATGGGGGCAA-3′.

Each 20 μL reaction contained 10 μL of master mix, 0.4 μM of each primer, and 2 μL of template DNA. The cycling conditions were: 95 °C for 2 min; 40 cycles of 95 °C for 5 s and 60 °C for 30 s. This was followed by melting-curve analysis (65–95 °C). Negative and positive controls were included in each run. Viral genome copy numbers were calculated using a standard curve generated by CFX Manager software Version 3.1.

### 2.9. Infectivity of 3D-Amplified Virus

Supernatants collected from 3D cultures at 3 dpi were used to infect 2D Vero cells at an MOI of 1. Cells and supernatants were harvested at 1, 2, and 3 dpi. Viral DNA was extracted and quantified by qPCR as described above.

### 2.10. Western Blot Analysis

For 3D cultures, gel–cell constructs were collected at 12 hpi, 1 dpi, 2 dpi, and 3 dpi. For 2D cultures, cells were harvested at 4, 8, 12, 20, 24, and 28 hpi.

Total protein (25 μg per sample) was separated by 12% SDS-PAGE and transferred onto Protran^®^ nitrocellulose membranes (Pall Corporation, New York, NY, USA). Membranes were blocked with 2% bovine serum albumin (BSA) for 2 h at room temperature and incubated overnight at 4 °C with primary antibodies against LC3B and p62 (R&D Systems, Minneapolis, MN, USA) and GAPDH (Signalway Antibody, College Park, MD, USA).

After washing, membranes were incubated with HRP-conjugated goat anti-rabbit IgG (Beyotime, Shanghai, China). Bands were visualized using enhanced chemiluminescence reagent (Advansta, Menlo Park, CA, USA). GAPDH served as the loading control. Band intensities were quantified using ImageJ 1.54 g (NIH, Bethesda, MD, USA).

### 2.11. Statistical Analysis

Data were analyzed using SPSS version 29.0 (IBM Corp., Armonk, NY, USA). The results are presented as the mean ± standard deviation (SD). Differences between two independent groups were evaluated using the independent-samples *t*-test. All experiments were performed in triplicate or more. A *p*-value < 0.05 was considered statistically significant.

## 3. Results

### 3.1. Scaffold Characterization of RADA16-I

**Figure 1:**
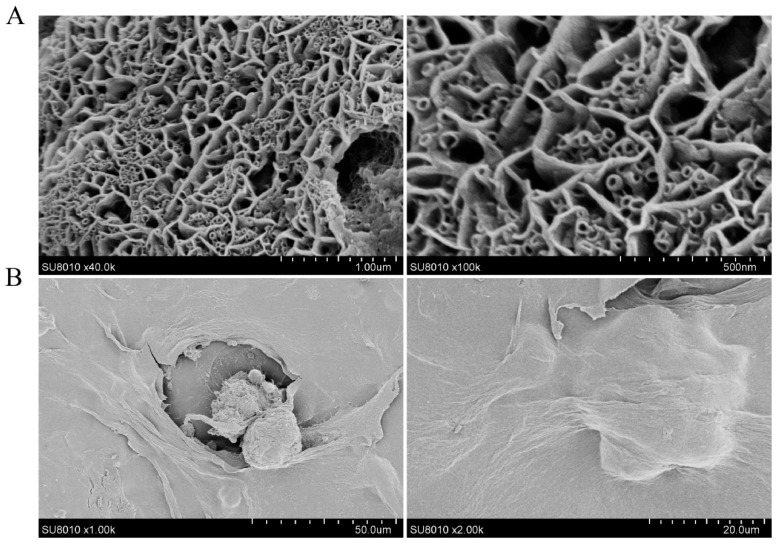
RADA16-I was characterized by SEM and subsequently used for 3D cell culture. (**A**) SEM micrographs revealed that RADA16-I peptides self-assembled into an interconnected network of nanofibers with a pore size comparable to that of native extracellular matrix. (**B**) Vero cells encapsulated within the RADA16-I formed well-defined cellular clusters.

Scanning electron microscopy (SEM) analysis of the nano-self-assembling peptide RADA16-I (Figure 1) revealed its capacity to self-assemble into nanofibers exhibiting a reticular architecture in ion-containing solutions. These nanofibers interwove to form a porous network with a pore structure conducive to the free diffusion of air and nutrients, akin to the porosity of the natural extracellular matrix (ECM). Furthermore, cells encapsulated and entangled within the nanofiber mesh demonstrated a 3D growth pattern.

### 3.2. Cell Growth in 3D Culture

**Figure 2:**
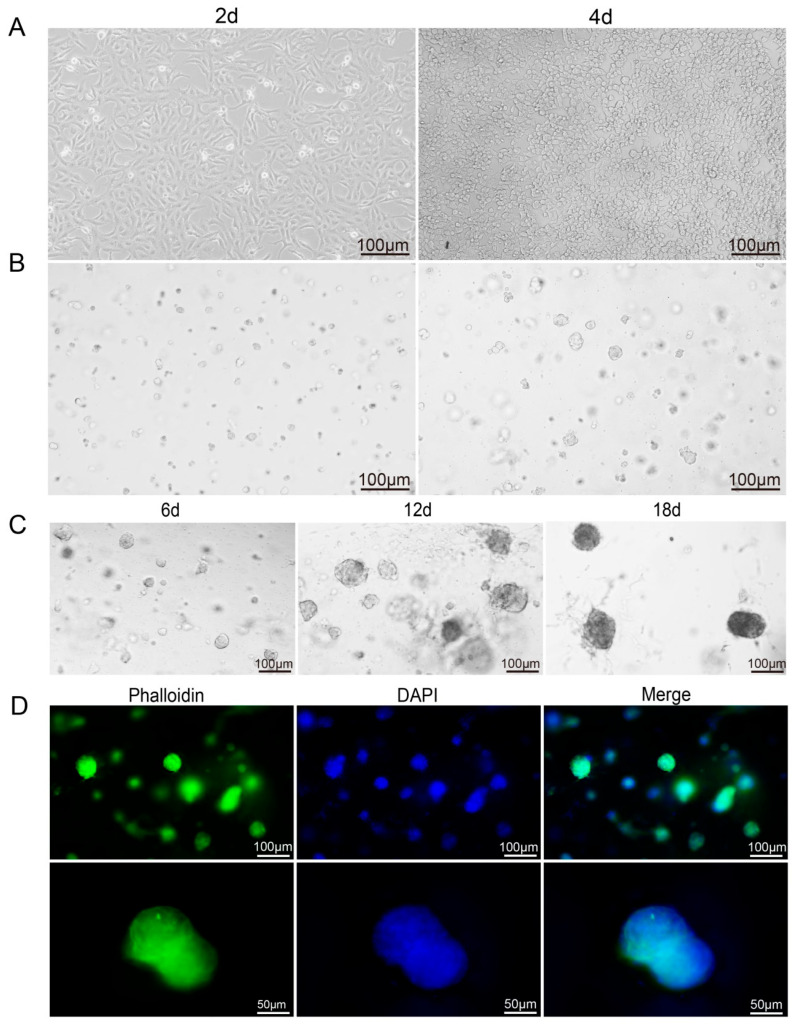
Vero cell growth in 2D and 3D culture. (**A**) Vero grown in 2D culture exhibit pike-shaped adherent growth, and by 4 d, the cells have partially rounded and detached. (**B**) Vero grown in 3D culture, growing in a ball of cell clusters with pseudopods protruding. (**C**) Vero cultured in RADA16-I peptide for 6 d, 12 d and 18 d; the cell clusters become larger and have pseudopods protruding around them as the culture time increases. (**D**) Phalloidin staining at day 18 of Vero cell culture with spherical cell growth. Phalloidin staining(green) shows the microfilament network, and nuclei were counterstained with DAPI (blue).

Growth difference between 2D and 3D culture: In 2D culture, Vero cells assumed a spindle-like morphology and adhered strongly to the substrates. By the 4th day, delayed passaging resulted in excessive proliferation, depleting nutrients and available surface area, which led to contact-induced compression, progressive deformation, and eventual rounding and detachment (Figure 2A). Conversely, in 3D culture, Vero cells initially existed as individual entities and subsequently aggregated to form multicellular spheroids, which became apparent on the 2nd day. As the culture period extended, the volume of these cell clusters continued to increase, and their surface characteristics underwent significant alterations: the initially smooth surface gradually became rough, with the emergence of distinct protrusions. Furthermore, two morphologies of Vero cells were observed in the 3D culture: the majority formed multicellular spheroids, while a minority exhibited a branching morphology. This dynamic process not only showed the unique growth of Vero cells in 3D culture but also suggested their distinctive morphological transformations (Figure 2B–D).

**Figure 3:**
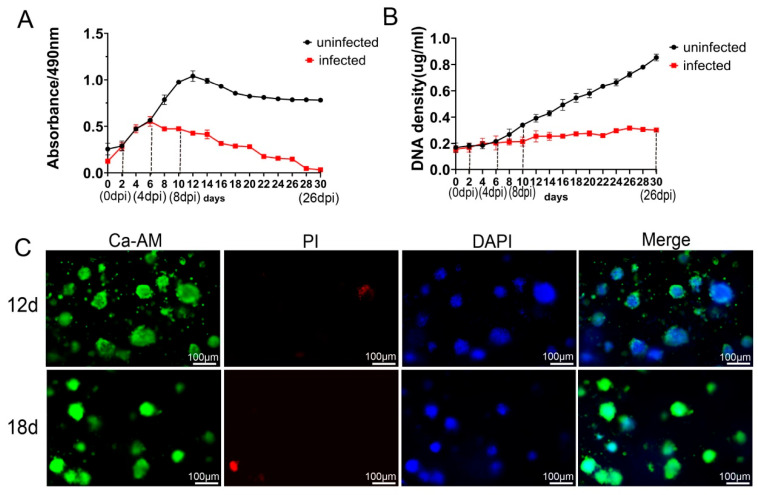
Vero activity and proliferation in RADA16-I 3D culture. (**A**) Vero growth activity in RADA16-I 3D culture and activity after infection with HSV-1 was assayed by MTS; the uninfected group exhibited increased activity with increasing incubation time, reaching a peak at day 12, and then maintained higher activity until day 30, while the infected group showed a gradual decrease in activity. (**B**) The proliferation of Vero in RADA16-I 3D culture and after infection with HSV-1 as measured by DNA quantification; uninfected cells continued to proliferate with increasing time in culture, whereas infection proliferation gradually decreased. (**C**) Ca-AM staining of Vero cultured for 12 and 18 days with strong green fluorescence (Ca-AM is green) and essentially no red fluorescence (PI is red), indicating good cell growth activity. DAPI staining (blue) shows cell nuclei.

To further evaluate the viability and proliferation of Vero cells within RADA16-I 3D culture, MTS assays and DNA quantification were performed. The results showed a progressive increase in both cell viability and proliferation with extended culture duration, with viability reaching its peak at 12 days. Notably, robust viability was sustained for up to 30 days, suggested that this model may be suitable for subsequent long-term infection experiments and could be employed to investigate persistent infection, latency, integration, and neurodegeneration (Figure 3A,B). Furthermore, Calcein-AM (Ca-AM) staining exhibited strong green fluorescence at 12 days, indicative of high cell viability, with minimal red fluorescence. By 18 days, an increase in red fluorescence was observed (Figure 3C), likely due to localized cell death within the cell clusters as a result of nutrient depletion in the interior regions.

### 3.3. Proliferative Activity of Vero Cells Infected with HSV-1 in the RADA16-I 3D Culture

**Figure 4:**
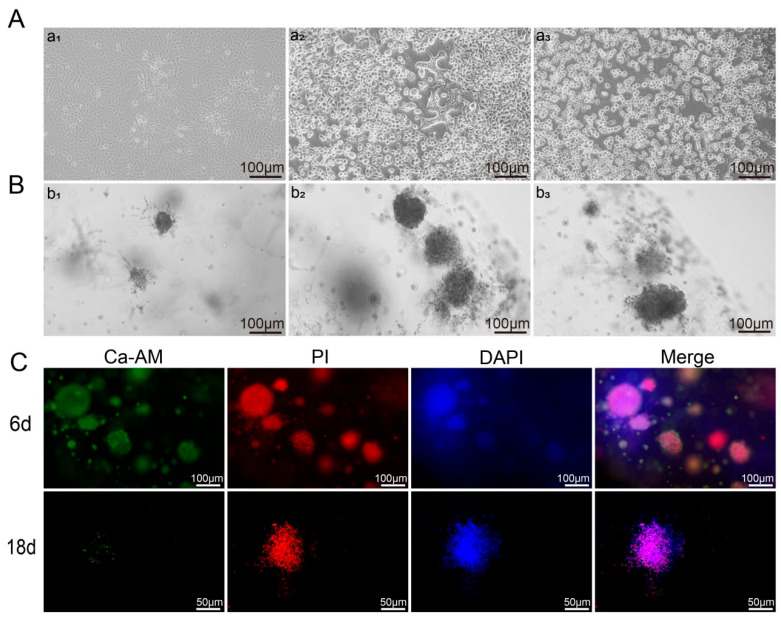
Changes in morphology and viability of cells infected with HSV-1 in Vero. (**A**) In 2D culture on 1 dpi (**a_1_**), 2 dpi (**a_2_**), and 3 dpi (**a_3_**), intercellular spaces widened, and cells became rounded and fragmented. (**B**) In 3D cultures on 6 dpi (**b_1_**), 12 dpi (**b_2_**), and 18 dpi (**b_3_**), cell clusters exhibited rough surfaces, underwent lysis, and were surrounded by scattered cellular debris. (**C**) Ca-AM staining for cell activity on 6 dpi and 18 dpi, with some green fluorescence on 6 dpi and weak green fluorescence on 18 dpi.Ca-AM staining (green) shows live cells, PI staining (red) shows dead cells, and DAPI staining (blue) shows cell nuclei.

Utilizing the established 3D cell model, the experiments confirmed the feasibility of employing the RADA16-I 3D model for long-term cell culture. Additionally, the model was infected with HSV-1 (MOI = 1) to investigate its capacity for viral propagation within a 3D environment. In 2D culture, HSV-1 infection induced notable morphological alterations in cells over a three-day period. On the 1st day post-infection (dpi), a minority of cells transitioned from a spindle-shaped to a rounded morphology, accompanied by the initial formation of intracellular vacuoles (Figure 4A(a_1_)). By the 2nd dpi, the majority of cells had adopted a rounded shape, surrounded by prominent cellular debris. Intercellular spaces expanded, and a substantial number of vacuolated, reticular cells were observed (Figure 4A(a_2_)). On the 3rd dpi, all cells exhibited a rounded morphology, with most detaching from the substrate and floating in the culture medium (Figure 4A(a_3_)). The culture environment was characterized by extensive cellular debris, indicative of pronounced cytolytic effects. In contrast, the 3D culture displayed distinct pathological manifestations compared to the 2D culture. At 3 dpi, the surface of the cell clusters already exhibited significant irregularities, suggesting early structural changes within the cells. By 6 dpi, the cell clusters began to disintegrate, with scattered individual cells and cellular debris becoming evident (Figure 4B(b_1_)). This suggested that the pathological changes initiated at the surface of the cell clusters and progressively spread inward, exacerbating with prolonged infection duration—consistent with previous reports [18]. Between 12 and 18 dpi, the cell clusters continued to disintegrate, with a marked increase in the number of individual cells and cellular debris (Figure 4B(b_2_,b_3_)). This dynamic progression of infection highlights the profound effect of HSV-1 on the structural organization of 3D cellular aggregates, as visually observed in our model system.

The viability and proliferation of cells following HSV-1 infection were further examined. In the RADA16-I 3D culture, samples collected at various time points were analyzed using DNA quantification, Ca-AM dyeing, and MTS assays. Compared to the uninfected controls, cell activity and proliferation were found to gradually decline after 2 dpi, with virtually no cell activity observed by 30 dpi (Figure 3A,B). Furthermore, observations of the 3D culture on 6 dpi and 18 dpi using Ca-AM staining revealed that although cells still exhibited some viability at 6 dpi, cell viability had significantly decreased by 18 dpi (Figure 4C). Our observations indicate successful HSV-1 infection within the RADA16-I-based 3D culture.

### 3.4. Replication of HSV-1 in RADA16-I 3D Culture

**Figure 5:**
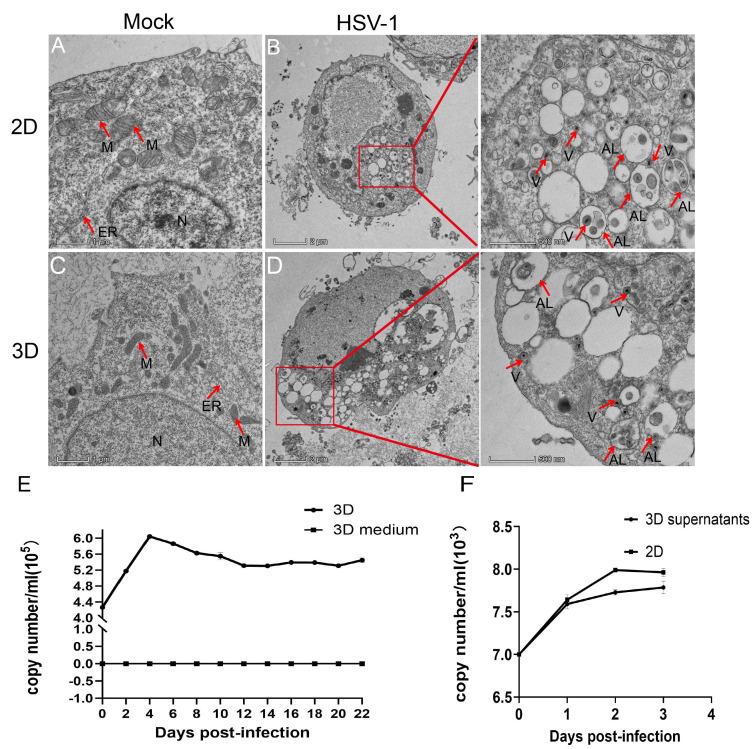
Observation of viral particles by TEM and qPCR to detect viral replication. (**A**) An uninfected Vero cell in 2D. (**B**) Vero cells infected with HSV-1 for 16 hpi in 2D. (**C**) An uninfected Vero cell in 3D. (**D**) Vero cells infected with HSV-1 for 3 dpi in 3D. M: mitochondrion; N: nucleus; ER: endoplasmic reticulum; V: virus; AL: autophagosome/autolysosome. (**E**) To understand the replication of HSV-1 in RADA16-I 3D culture by qPCR, the virus replication peaked on 4 dpi, and then maintained a certain level of replication until 22 days. (**F**) qPCR measurements for 3D replicated HSV-1 infectivity and for 2D culture with infectious HSV-1 replication. 3D supernatants are supernatants collected after 3 dpi of 3D HSV-1 infection followed by infection of 2D Vero, and then collection of supernatants and cellular qPCR amplification.

Cell viability and phenotypic alterations subsequent to HSV-1 (MOI = 1) infection are shown in the 3D model above. TEM was utilized to further investigate the viral particles within infected cells, with the aim of revealing ultrastructural changes. Notably, mitochondria and the endoplasmic reticulum exhibited pronounced swelling, nuclei were ruptured, and numerous vacuoles were observed in the cytoplasm. Additionally, viral particles and autophagy-related vesicles were identified within the cells. The presence of viruses and autolysosomes/autophagosomes was observed in both 2D and 3D cultures (Figure 5A–F). Further analysis of viral replication in RADA16-I 3D culture using absolute quantitative PCR (qPCR) indicated that the viral load peaked at 6.03 × 10^5^ copies/mL on 4 dpi and subsequently declined gradually, yet continued to propagate for up to 22 days. To eliminate the potential influence of viral DNA in the hydrogel on experimental outcomes, HSV-1 infection analysis was conducted on cell-free hydrogels, with the results indicating no detection of viral DNA in either the hydrogel or the supernatant. Moreover, the qPCR results suggested that viruses amplified in 3D culture remained infectious, consistent with the trend of viral replication in 2D culture (Figure 5E,F). These results collectively support that the RADA16-I-based 3D model sustains active HSV-1 replication and yields infectious viral progeny.

### 3.5. Autophagy in HSV-1-Infected Vero Cells Differed Between 2D and 3D Models

In the context of our 3D model, the morphological alterations following HSV-1 infection were suggestive of potential intracellular stress and possible engagement of autophagy-related pathways. To further characterize the cellular response, we proceeded to analyze the expression and processing of key autophagy-related markers in both culture systems. Samples of HSV-1 (MOI = 1)-infected Vero cells were collected at various time points under both 2D and 3D culture conditions, and the abundance levels of autophagy-related proteins P62 and LC3B-II/LC3B-I were detected using Western blot analysis in relation to HSV-1 expression levels post-infection.

**Figure 6:**
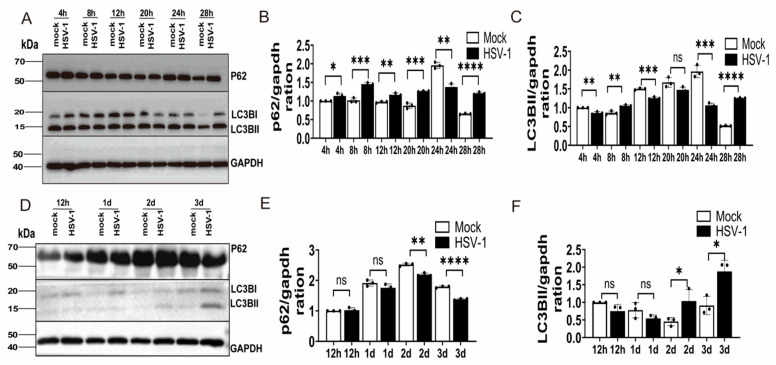
Time-dependent autophagy after HSV-1 infection in 2D culture and spatially dependent autophagy after HSV-1 infection in 3D culture. (**A**) Two-dimensional Vero cells uninfected (mock) and infected with HSV-1(HSV-1) at an MOI of 1 were analyzed at 4, 8, 12, 20, 24 and 28 hpi. (**B**) P62 and (**C**) LC3BII protein levels were normalized to GAPDH. (**D**) Three-dimensional Vero cells uninfected (mock) and infected with HSV-1(HSV-1) at an MOI of 1 were analyzed at 12 hpi, 1 dpi, 2 dpi, 3 dpi. (**E**) P62 and (**F**) LC3BII protein levels were normalized to GAPDH. Statistical significance was determined using the independent samples *t*-test. The bars represent the mean ± SD of biological replicates (p62 n = 3; LC3BII /LC3BI = 3); ns: not significant; * *p* < 0.05; ** *p* < 0.01; *** *p* < 0.001; **** *p* < 0.0001.

In 2D culture, following HSV-1 infection of Vero cells, P62 protein levels increased from 4 hpi to 20 hpi, whereas LC3B-II levels exhibited only a transient increase at 8 hpi, followed by a subsequent decline. Overall, HSV-1 infection appeared to inhibit the cellular autophagy process at this stage. However, both P62 and LC3B-II levels decreased after 24 hpi, potentially suggesting that autophagosomes accelerated their progression into the lysosome for degradation, thereby facilitating the completion of autophagy flux. Conversely, after 28 hpi, both P62 and LC3B-II levels rose significantly, suggesting an impediment in the degradation process of autophagosomes, which in turn inhibited the normal progression of autophagy flux (Figure 6A–C). Under our experimental conditions, the autophagic response to HSV-1 infection in 2D monolayers appears to be regulated in a time-associated manner.

In 3D culture, the alterations in cellular autophagy post-HSV-1 infection exhibited distinct characteristics. At 12 hpi and 1 dpi, the levels of autophagy-related proteins P62 and LC3B-II remained largely unchanged. Given this, we reasoned that autophagy was not significantly activated. However, on 2 dpi and 3 dpi, P62 levels decreased significantly, while LC3B-II levels increased markedly. This pattern of marker changes leads us to speculate that HSV-1 infection might concurrently facilitate autophagosomes’ biogenesis and their subsequent lysosomal degradation, potentially upregulating overall autophagic flux. This hypothesis, illustrated in (Figure 6D–F), requires further experimental validation.

## 4. Discussion

In molecular studies of HSV-1, 2D cell culture is widely used as a foundational experimental model due to its simplicity and ease of experimental control [19,20]. However, this approach has well-recognized limitations, particularly its inability to recapitulate the complex in vivo microenvironment and its relatively short culture lifespan, which restricts modeling of long-term physiological or pathological processes [21]. In the present study, Vero cells cultured under 2D conditions exhibited a spindle-like growth pattern. In the absence of passaging, partial cell death and detachment occurred by day 4, likely due to spatial constraints and nutrient depletion. These findings are consistent with our previous observations in 293T cells, which similarly displayed flattened morphology and a limited culture duration under 2D conditions [18]. In contrast, Vero cells cultured in RADA16-I-based 3D systems formed aggregates with two distinct morphological phenotypes. This heterogeneity may reflect differences in metabolic state, proliferative capacity, or susceptibility to viral infection [22]. Notably, although total cellular DNA content increased over time, MTS-determined viability plateaued after day 12. We speculate that the continuous enlargement of spheroids leads to hypoxia or nutrient depletion in the central region, or that due to cellular respiration, the oxygen concentration surrounding cells (pericellular oxygen) is considerably lower than the gas-phase oxygen concentration set in the incubator, thereby weakening metabolic activity [23,24]. Importantly, the majority of cells organized into compact spheroids that maintained robust viability for up to 30 days, consistent with previous reports [25]. Within this 3D microenvironment, renal epithelial-derived Vero cells supported sustained, non-lytic HSV-1 infection under conditions of aggregate architecture, high cell density, and metabolic stress. These findings suggest that HSV-1 may possess a broader cellular capacity for long-term maintenance than previously appreciated. This observation is in line with the work of Cohen et al. [26], who reported that abortive HSV-1 infection can induce spontaneous latency-like quiescence in non-neuronal cell populations. Collectively, our results support the use of this 3D system as a physiologically relevant in vitro platform for investigating HSV-1 persistence in non-neuronal CNS reservoirs, including astrocytes and oligodendrocytes.

The relationship between HSV-1 infection and autophagy remains complex and, in some contexts, controversial. For example, studies in HEK-293T cells demonstrated that the HSV-1 tegument protein UL21 can induce selective autophagy via the cargo receptor TOLLIP [19]. Similarly, infection of glioma cells and keratinocytes has been shown to trigger early autophagy-dependent restriction of viral replication [27]. Conversely, HSV-1 encodes multiple factors that suppress or modulate autophagy, thereby promoting viral replication and immune evasion [28,29]. Thus, the autophagic response to HSV-1 is highly context-dependent, varying with cell type, infection stage, and microenvironmental conditions.

In our 2D model, HSV-1 infection elicited a time-dependent biphasic autophagic response. Autophagy-related markers were initially suppressed, followed by marked upregulation at later stages of infection. Transmission electron microscopy confirmed the accumulation of autophagic vesicles following infection. The early suppression phase is consistent with prior reports [30] and may reflect a viral strategy to transiently modulate the host intracellular environment to favor early replication. The subsequent increase in autophagic activity likely represents a shift in host–pathogen dynamics, potentially facilitating antiviral defense. Indeed, autophagy has been shown to restrict HSV-1 replication through degradation of viral proteins, including ICP34.5 [31].

The influence of culture dimensionality on cellular behavior is well established. Our previous work demonstrated distinct drug sensitivity profiles between 2D and 3D cultures, with 3D systems exhibiting enhanced physiological relevance [32]. Baseline autophagy levels have likewise been shown to depend intrinsically on culture architecture [33]. In the present study, we observed striking differences in autophagic dynamics between 2D monolayers and 3D spheroids following HSV-1 infection.

In the 3D model, the onset of detectable autophagic activity was notably delayed. From 12 h post-infection (hpi) to 1 day post-infection (dpi), no significant changes were observed in autophagy markers, suggesting maintenance of autophagic homeostasis. This delay may result from the physical barrier imposed by the extracellular matrix, which could limit viral penetration, or from enhanced intercellular junctional integrity that modulates viral receptor accessibility. By 2 dpi, autophagic flux was robustly activated. This activation may be linked to enhanced cellular polarity and lysosomal maturation in 3D culture—potentially reflected by increased LAMP1 expression [34]—or to restricted viral replication that diminishes effective immune evasion. At 3 dpi, autophagy-related indices remained elevated, suggesting that autophagy-mediated viral clearance had become a dominant process. Mechanistically, 3D architecture may potentiate autophagy–lysosome function through mechanical signaling pathways, such as YAP/TAZ inhibition, or promote partial viral genome silencing [35]. Importantly, the temporal increase in autophagy preceded the peak of viral replication (4 dpi by qPCR), aligning with the activation window of cell-intrinsic antiviral defenses. This temporal sequence underscores the value of the RADA16-I 3D microenvironment for modeling staged host–pathogen interactions and provides a more physiologically grounded framework for studying HSV-1–autophagy interplay.

Overall, we established a long-term 3D HSV-1 infection model using RADA16-I hydrogel and demonstrated that HSV-1 can sustain replication within this system. Moreover, the host autophagic response differed fundamentally between 2D and 3D contexts. In 2D culture, autophagy exhibited a biphasic pattern consistent with progressive viral modulation. In contrast, the 3D microenvironment was associated with delayed yet sustained autophagic activation, highlighting the critical influence of tissue-like architecture on antiviral defense mechanisms.

Several limitations merit consideration. First, although Vero cells represent a classical and highly permissive model for HSV-1 research, their deficiency in type I interferon production may result in autophagy regulation that differs substantially from that of human cells. Future studies aimed at dissecting host defense mechanisms should incorporate more physiologically relevant systems, such as primary human neurons, mouse embryonic fibroblasts, dendritic cells, or glial cell lines, which more accurately recapitulate cell-type-specific autophagy networks. Second, mechanistic depth should be enhanced by validating autophagy-related findings at the genetic level, including CRISPR-mediated knockdown approaches and dual-fluorescence autophagic flux reporters in both 2D and 3D systems. Third, regarding viral penetration heterogeneity in 3D cultures, the dense multicellular spheroids formed in RADA16-I scaffolds inherently limit uniform virus distribution. Future studies could employ microfluidic-based pore size control and magnetic manipulation [36] to optimize scaffold architecture and facilitate more homogeneous viral penetration.

## 5. Conclusions

We developed a 3D culture model using the self-assembling peptide RADA16-I as a scaffold. This system enabled sustained HSV-1 replication and may serve as a platform to study prolonged infection phases. Comparative analysis further identified distinct patterns in the autophagic response between two-dimensional and three-dimensional environments.

## Data Availability

The original contributions presented in this study are included in the article. Further inquiries can be directed to the corresponding authors.

## Abbreviations

2D	two-dimensional
3D	three-dimensional
Ca-AM	calcein acetoxymethyl ester
dpi	days post infection
ECM	extracellular matrix
hpi	hours post infection
HSV-1	herpes simplex virus type 1
MEM	minimum essential medium
PBS	phosphate-buffered saline
qPCR	real-time quantitative polymerase chain reaction
SDS-PAGE	sodium dodecyl sulfate–polyacrylamide gel electrophoresis
SEM	scanning electron microscope
TEM	transmission electron microscope
WB	Western blot
